# Higher Sensitivity and Earlier Identification of Celiac Disease Autoimmunity by a Nonradioactive Assay for Transglutaminase Autoantibodies

**DOI:** 10.1155/2016/2904563

**Published:** 2016-12-26

**Authors:** Zhiyuan Zhao, Dongmei Miao, Kathleen Waugh, Iman Taki, Fran Dong, Edwin Liu, Marian Rewers, Yu Liu, Liping Yu

**Affiliations:** ^1^Department of Endocrinology, 2nd Hospital of Jilin University, Changchun, Jilin, China; ^2^Barbara Davis Center for Childhood Diabetes, University of Colorado Denver, Aurora, CO, USA; ^3^University of Colorado Children's Hospital, Aurora, CO, USA; ^4^Sir Run Run Hospital, Nanjing Medical University, Nanjing, China

## Abstract

Higher sensitive transglutaminase autoantibody (TGA) assay will detect the onset of celiac disease (CD) autoimmunity earlier. In developing a nonradioactive assay for TGA, we utilized electrochemiluminescence (ECL) technology and compared it to a high-performance radioimmunoassay (RIA) currently being used to screen patients with type 1 diabetes (T1D) and genetically at-risk individuals for CD. We selected 183 T1D patients with 60 patients having received biopsy and analyzed 396 sequential samples from 73 young children longitudinally followed up with TGA seroconversion, with 27 undergoing biopsy. In addition, 112 age-matched healthy control subjects were included in the study. With the 99th percentile of specificity, the ECL assay detected significantly more TGA positivity among patients with T1D (133/183) than RIA (114/183) and more of the sequential samples (34%) from 73 children than RIA (18%). The TGA assay performed by ECL was positive in all 59 subjects with villous atrophy. Among 73 longitudinally followed up children, ECL assay had earlier detection of TGA on 34 children by a mean of 2.5 years. In conclusion, the new TGA assay by ECL has a higher sensitivity than the current RIA assay and may better predict the onset of CD.

## 1. Introduction

Celiac disease (CD) is well defined as a chronic small intestinal autoimmune enteropathy precipitated by exposure to dietary gluten in genetically predisposed individuals. The prevalence of CD is very high with screening studies suggesting an increasing frequency from 0.3% up to 3% [1–6]. The clinical presentation of CD is highly variable and targeted screening based on clinical symptoms is not effective in diagnosing the majority of individuals with CD in the general population [7]. Therefore, many individuals remain undiagnosed.

Determination of autoantibodies to tissue transglutaminase (TGA) is currently the most effective single serologic test for the identification of CD or the development of autoimmunity associated with CD [8]. Routine screening is recommended for individuals considered to be at high risk, including those with type 1 diabetes [9] and those with a family member with CD among others [10]. Very recently, a successful measurement of TGA in saliva samples was reported [11] that would be a convenient way for large cohort screening, especially among young children. In 2009, a TGA workshop with a large international collaborative effort toward improving and standardizing TGA measurement was organized [12]. The workshop found that radioimmunoassays (RIAs), in general, were more quantitative and more sensitive than standard ELISA assays in detecting low-titer sera (including serially diluted samples). Such assays with higher sensitivity allow detection of the earliest signs of the development of CD autoimmunity, which is critical for such studies investigating environmental triggers of autoimmunity such as in the Diabetes Autoimmunity Study in the Young (DAISY) [13] and the large international clinical trial, the Environmental Determinants of Diabetes in the Young (TEDDY) [14]. However, the RIA for TGA is not widely accepted due to the necessity of radioactivity and limited access to laboratories that can perform this assay, as opposed to currently utilized solid-phase assays. We have recently developed and extensively validated nonradioactive islet autoantibody assays for type 1 diabetes using electrochemiluminescence (ECL) detection with excellent sensitivity and specificity compared to the current standard RIAs for islet autoantibody measurements [15–18]. In the present study, we developed a nonradioactive TGA assay with ECL technology using a similar assay format as for islet autoantibody measurement. We analyzed TGA by ECL in 183 selected patients with type 1 diabetes who had the TGA measured by RIA and 396 sequential samples from 73 children who were longitudinally followed up with TGA seroconversion. The comparisons with current standard RIA for assay sensitivity, specificity, and the time of TGA seroconversion were analyzed.

## 2. Research Design and Methods

### 2.1. Definitions

In this study, CD is defined as having an intestinal biopsy showing a Marsh score of 2 or greater by original Marsh criteria [19]. CD autoimmunity is defined as having persistent TGA positivity on 2 or more sequential measurements done over time.

### 2.2. Subjects

The serum samples were from 183 patients with type 1 diabetes who were followed up having routinely TGA screened by radioimmunoassay (RIA) at the Barbara Davis Center. The patient ages were ranged from 2.2 to 41.7 years with median age of 13.0 and 51% were female (94/183). Most patients with TGA positivity were positive on more than one occasion, and 60 patients had an intestinal biopsy. Of these, 42 were diagnosed with CD. In addition, we analyzed 73 subjects from the DAISY birth cohort (having a genetic risk for CD) having a total of 396 serial samples obtained longitudinally over a period of 15 years. TGA positivity was identified in all of them by RIA at some point during the follow-up. Twenty-seven of these subjects were biopsied during their follow-up and 17 were diagnosed with CD based on biopsy findings. Of the 112 gender-matched normal control samples tested, all were negative for TGA by RIA. Signed written informed consents were obtained from participants and the study was approved by the Institutional Review Board of the University of Colorado.

### 2.3. ECL-TGA Assay

The method of the ECL-TGA assay was adopted from the format of an ECL-GADA assay previously published [17]. The interaction of antibodies in sera with labeled antigen was completed in the liquid phase. One transglutaminase antigen labeled with the biotin allows capture on the streptavidin coated solid phase. The other transglutaminase antigen with the Sulfo-tag provides electrochemical light emission for detection of the captured complex. Both the biotinylated and Sulfo-tagged transglutaminase were used as competitors and tested in our standard RIA. Both modified molecules were able to compete well with S-35 transglutaminase for binding to TGA in patient sera. Following a series of optimization steps, the ECL assay protocol described below was used for all experiments. In brief, 4 *µ*l of serum premixed with 16 *µ*l of PBS buffer was incubated with 20 *µ*l of antigen buffer containing both Sulfo-tag labeled transglutaminase protein (DIARECT AG, Freiburg, Germany) at the concentration of 100 ng/ml and biotin-labeled transglutaminase protein at the concentration of 400 ng/ml in PBS containing 5% BSA for overnight at 4°C. On the 2nd day, 30 *µ*l of overnight incubates was added per well onto a streptavidin coated plate (MSD, Gaithersburg, MD) and incubated for 1 hour at room temperature. After 3 rounds of washing, the plate was counted on an MSD counter, Imager 2400 (MSD, Gaithersburg, MD). The results were expressed as an index against our internal standard positive control serum, the same standard positive control serum used for the RIA. The assay upper limit of normal range (index 0.015) was set at >99th percentile of 112 normal control samples that were characterized as TGA negative by RIA. The interassay coefficient variations (CV) were 8.1% (*n* = 30) with index value around 1.0 and 16.2% (*n* = 30) with index value around 0.05.

### 2.4. RIA-TGA

The method of RIA-TGA was published previously [20] and the upper limit of normal (index 0.050) was established as the 100th percentile of 184 healthy control subjects. The interassay CV for the sample was 8.9% (*n* = 250) with index value around 1.0 and 19.3% (*n* = 15) with index value around 0.09.

### 2.5. Statistics

Statistical analyses were performed using correlation analysis for TGA levels of two assays, McNemar's test for comparing the sensitivity, with PRISM 4.0 version software (GraphPad Software Inc., San Diego, CA). A two-tailed *p* value with an alpha level for significance was set at 0.05.

## 3. Results

### 3.1. Assay Sensitivity Titration

**Figure 1:**
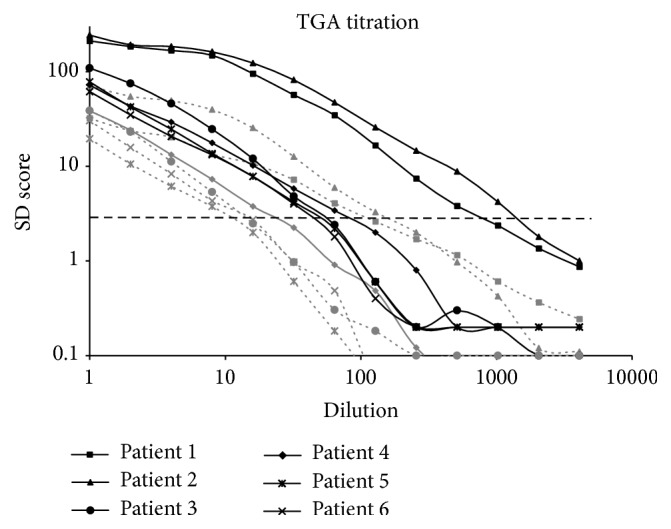
Titration of TGA by both RIA and ECL assay using a TGA positive sample from six of T1D patients studied with confirmed clinical CD by biopsy (marked patient 1, patient 2, patient 3, patient 4, patient 5, and patient 6, respectively). The samples were in a serial of 1 : 1 dilution with a normal control serum to a maximum of 1 : 4096 dilutions. The solid curves were TGA levels from ECL assay and the dotted curves with gray color were TGA levels from RIA. A horizontal dotted line represents assay cut-offs for both ECL and RIA at 3 standard deviation (SD) scores. The SD scores are calculated with the following formula: (index value − mean index of controls)/SD.

To titrate the sensitivity of ECL-TGA assay and compare it with a current high-performance RIA-TGA assay, 6 TGA positive samples from 6 patients studied, respectively, with confirmed clinical CD by biopsy were in a serial of 1 : 1 dilution with a normal control serum to a maximum of 1 : 4096 dilution and measured for TGA by both ECL and RIA. The levels of TGA titrated with ECL and RIA were both plotted in Figure 1 with solid line (dark color) for ECL and dotted line (gray color) for RIA, respectively. The last titration points showing TGA positive for RIA versus ECL were 1 : 64 versus 1 : 512 for patient 1, 1 : 128 versus 1 : 1024 for patient 2, 1 : 8 versus 1 : 32 for patient 3, 1 : 16 versus 1 : 64 for patient 4, 1 : 8 versus 1 : 32 for patient 5, and 1 : 8 versus 1 : 32 for patient 6, which clearly demonstrated that TGA measured by ECL method was much more sensitive than that by RIA.

### 3.2. Higher Sensitivity of ECL Than RIA in Detecting TGA in Patients with Type 1 Diabetes

**Figure 2:**
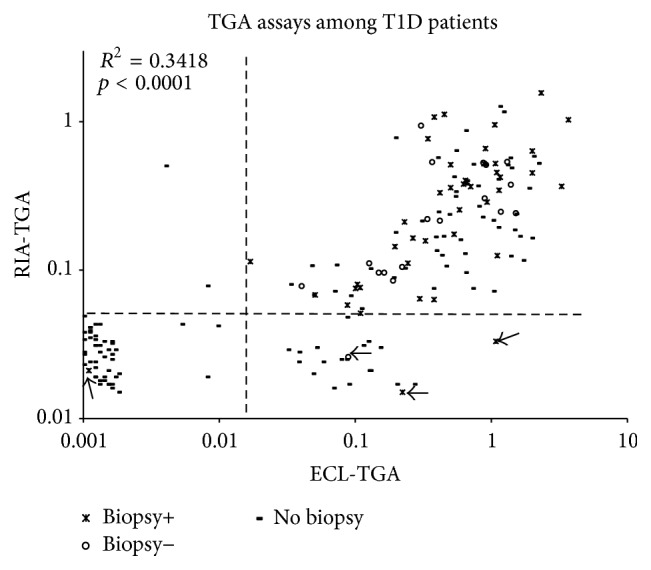
TGA levels from ECL assay and our current standard RIA were compared among 183 patients with type 1 diabetes. The cases without or with biopsy+ or − are shown in different markers. Four cases from biopsy group that became TGA negative by RIA were pointed by arrows.

With similar assay specificity set for both assays among healthy controls, the ECL assay detected significantly more TGA positivity among patients with type 1 diabetes (73%, 133/183) than RIA (62%, 114/183; *p* = 0.04). The levels of TGA between ECL and RIA as shown in Figure 2 correlated well (*R*^2^ = 0.3418, *p* < 0.0001). Of the 183 patients, 60 received intestinal biopsy upon their positive TGA results at the time by RIA and 42 were found biopsy positive. Many samples studied from these 60 biopsy patients were months to years after biopsy and the levels of TGA were found gradually declined during the follow-up (data not shown). Four samples in the present study from 3 patients with positive biopsy and one with negative biopsy became TGA negative by RIA (arrow-pointed in Figure 2) and dropped below the assay cut-off while ECL-TGA were still detectable for 3 of these 4 patients, 2 biopsy positive and one biopsy negative, and rest of biopsy patients were all positive for ECL-TGA. Compared between biopsy positive and negative subjects studied, the levels of TGA in mean values had no significant differences with both ECL (0.77 ± 0.83 versus 0.58 ± 0.50; *p* = 0.57) and RIA (0.37 ± 0.35 versus 0.29 ± 0.24; *p* = 0.79).

### 3.3. Earlier Detection of TGA by ECL Than RIA in Longitudinally Followed Children

**Figure 3:**
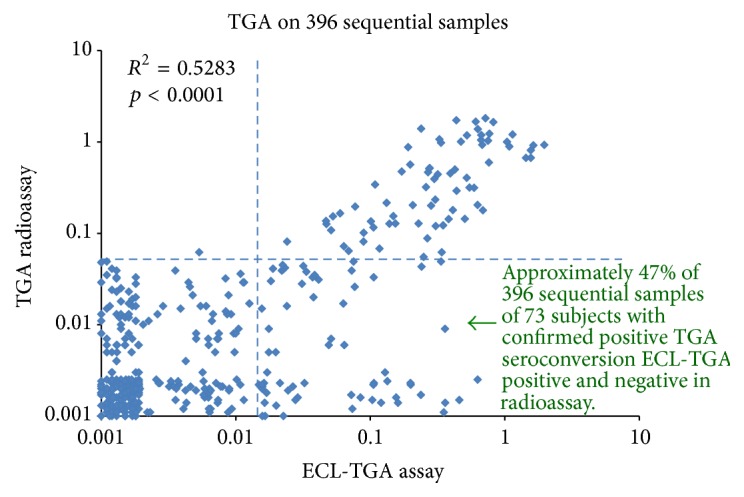
TGA levels from ECL assay and our current standard RIA were compared on 396 sequential samples from 73 children who were confirmed TGA positive seroconversion during the longitudinal follow-up.

**Figure 4:**
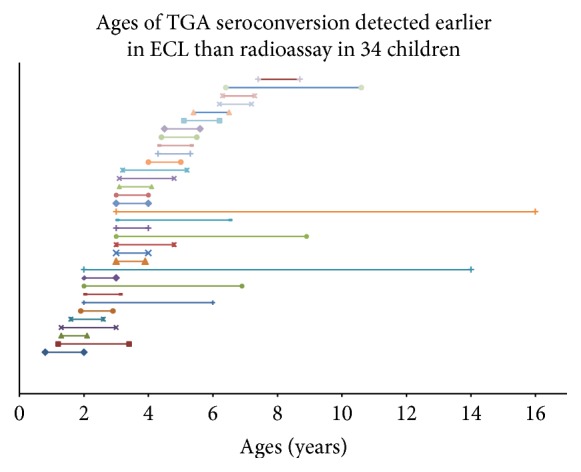
TGA were detected earlier in age in 34 children with ECL assay than RIA. The beginning of each line is TGA detecting age with ECL assay for a child and the end of line is TGA detecting age with RIA.

The group of 396 sequential samples from 73 children from DAISY who were longitudinally followed was tested using the ECL assay. These individuals were confirmed to have been TGA positive by RIA at some point in their follow-up. The levels of TGA between ECL and RIA were well correlated (*R*^2^ = 0.5281, *p* < 0.0001) as shown in Figure 3. TGA was detected in a total of 136 of the 398 sequential samples by ECL even though 64 of these 136 (47%) were undetectable by RIA. Among these 73 children with confirmed TGA positive seroconversion during the follow-up, ECL detected seroconversion earlier in 34 children when compared to RIA, identifying the onset of CD autoimmunity in these children by a mean of 2.5 years (0.8 to 13 years) earlier (Figure 4). In those 17 out of 27 with biopsy-confirmed disease, ECL detected TGA earlier than the RIA in 10 of these children by a mean of 2.1 years (range 0.8 to 6 years). The other 7 biopsy positive children detected TGA at the same clinical visit times by both ECL and RIA.

## 4. Discussion

The use of TGA assays with even greater sensitivity is invaluable in longitudinal cohort studies that follow the natural history of the development of CD autoimmunity. In particular, earlier detection of an immunologic change relevant to CD helps identify potential environmental triggers for the development of autoimmunity by reducing the apparent “lag time” between the two events during analysis. The gold standard for which ECL was compared to the RIA in this study. In children who are undergoing screening for CD because of an increased genetic risk (such as having type 1 diabetes or a family member with CD), autoantibody positivity often precedes the development of intestinal injury. Therefore, highly sensitive assays are utilized in this setting, and in clinical research, to detect the earliest signs of CD autoimmunity, which may lead to CD. It is clearly shown that different organ-specific autoimmune diseases are greatly overlapped. CD and type 1 diabetes share the high-risk HLA Class II of DR3-DQ2 and DR4-DQ8, and non-HLA genetic susceptibility in Caucasian population and their clinical phenotypes overlap in up to 10% of the patients [20]. In DAISY and TEDDY, all study participants are screened for CD autoimmunity by testing for TGA at least yearly. Persistent TGA positivity and CD are major endpoints in both studies [21, 22].

This TGA assay, performed by ECL, was modified from techniques currently utilized to measure islet autoantibodies. One reason why this assay may be superior to RIA in detecting the presence of TGA is because all immunoglobulin subclasses are able to react with the transglutaminase antigen and therefore could be captured in the ECL assay format (including IgA, IgG, IgM, or even IgE and IgD). In comparison, RIA detects only immunoglobulin subclass, IgA. In addition, when measuring islet autoantibodies, the ECL assay detects the more disease-specific high-affinity, high-risk autoantibodies [15, 17, 18] compared to RIA which detects more low-affinity, low-risk autoantibodies. This feature of disease specificity for TGA will need to be explored in CD.

It has been previously reported that higher TGA levels correlate better with villous atrophy [23]; assays performed by RIA generally have superior quantitative ability when measuring and reporting TGA. In the present group of patients studied, there were no correlations of higher TGA levels with positive intestinal biopsy found for both RIA and new ECL assay. It might be because many samples in the present study were months to years after biopsy and they will not reflect their actual TGA levels at the time of biopsy. Further study will be needed in determining the dynamics of CD autoimmunity and exploring the disease specificity using this new highly sensitive TGA assay.
